# Low-resistivity, high-resolution W-C electrical contacts fabricated by direct-write focused electron beam induced deposition

**DOI:** 10.12688/openreseurope.15000.1

**Published:** 2022-08-25

**Authors:** Pablo Orús, Fabian Sigloch, Soraya Sangiao, José María De Teresa

**Affiliations:** 1 Instituto de Nanociencia y Materiales de Aragon (INMA), Universidad de Zaragoza-CSIC, Zaragoza, 50009, Spain; 2 Laboratorio de Microscopias Avanzadas (LMA), Universidad de Zaragoza, Zaragoza, 50018, Spain

**Keywords:** electrical contacts, nanofabrication, focused electron beam induced deposition, superconductivity, transmission electron microscopy

## Abstract

**Background**: The use of a focused ion beam to decompose a precursor gas and produce a metallic deposit is a widespread nanolithographic technique named focused ion beam induced deposition (FIBID). However, such an approach is unsuitable if the sample under study is sensitive to the somewhat aggressive exposure to the ion beam, which induces the effects of surface amorphization, local milling, and ion implantation, among others. An alternative strategy is that of focused electron beam induced deposition (FEBID), which makes use of a focused electron beam

instead, and in general yields deposits with much lower metallic content than their FIBID counterparts.

**Methods**: In this work, we optimize the deposition of tungsten-carbon (W-C) nanowires by FEBID to be used as electrical contacts by assessing the impact of the deposition parameters during growth, evaluating their chemical composition, and investigating their electrical response.

**Results**: Under the optimized irradiation conditions, the samples exhibit a metallic content high enough for them to be utilized for this purpose, showing a room-temperature resistivity of 550 μΩ cm and maintaining their conducting properties down to 2 K. The lateral resolution of such FEBID W-C metallic nanowires is 45 nm.

**Conclusions**: The presented optimized procedure may prove a valuable tool for the fabrication of contacts on samples where the FIBID approach is not advised

## Plain language summary

We describe a new method to fabricate high-resolution, highly-conductive electrical contacts at the nanoscale based on a direct-write technique called focused electron beam induced deposition (FEBID). A W(CO) _6_ precursor and a scanning electron microscope are used for that aim. By optimizing the growth conditions we are able to create contacts with lateral dimension as small as 45 nm and, as a proof of concept, we grow contacts on a superconducting nanowire and measure its electrical properties down to very low temperature, 2 K. This means that our strategy works across a broad temperature range, from room temperature down to 2 K. Our work demonstrates that FEBID is a viable technique to make electrical contacts at the nanoscale without the burdens of the use focused ion beams, which in general create a lot of damage on the samples of interest. We plan to use FEBID to electrically contact materials of interest that are ion-sensitive, such as bidimensional materials, high-mobility semiconducting nanowires and oxides.

## Introduction

Measuring the electrical response of a sample or a device represents one of the most utilized and required characterization procedures in materials science and condensed matter physics, with different experiments ranging from conventional voltage-current characteristics to magneto-resistance studies ^1^, gating experiments ^2^, and many more. No matter how simple or complex the electrical configuration might be, having suitable contacts to electrically contact the nano- or micro-sized object with the macro-world is always a primary requirement for electrical properties characterization studies. As such, developing, employing, and improving nanopatterning techniques to adequately fabricate these contacts represents a parallel research field that is equally important to the investigation of the materials themselves ^3– 7^.

Examples of nanopatterning techniques that are commonly used for this purpose include optical lithography (OL) ^8^, electron beam lithography (EBL) ^9^, and focused electron/ion beam induced deposition (FEBID/FIBID) ^10, 11^. Both OL and EBL are resist-based lithography techniques – a radiation-sensitive spin-coated film (photosensitive and electron-resistive, respectively) is placed on top of the sample under study, and is then selectively exposed to the corresponding radiation. In OL, the exposure to ultraviolet radiation is performed through a mask, which allows for patterning of large areas, while in EBL the resist is exposed to electrons by means of a focused electron beam (FEB) that is scanned over the areas of interest ^9^. The resist is later removed in development and selective etching steps. On the other hand, in FIBID and FEBID, the deposition of the contacting material is achieved by injecting a gaseous precursor material containing the element of interest in close proximity to the sample, and then inducing its local decomposition by selectively scanning the corresponding beam over it ^10, 11^.

Contrary to OL and EBL, both FIBID and FEBID are single-step techniques: no resist is used to perform them, and no further steps are required after beam exposure ^10, 11^. In addition, the beam can be freely steered to trace patterns defined by the user without adding complexity to the procedure, which gives the technique an added value in the form of enhanced patterning flexibility. On the other hand, their serial nature also limits their applicability to smaller-scale contacting (in the order of µm), making them more fitting for research purposes and prototyping than for industrial applications other than circuit edit ^12^ and mask repair ^13^. Still, recent developed strategies based on the irradiation at cryogenic temperatures, which results in an enhancement of the growth rate by several orders of magnitude, point towards potential applicability of FIBID in the mm range ^14^.

FIBID is commonly implemented making use of a focused ion beam (FIB) of Ga ^+^ ions, owing to the commercial availability and ease of use of such systems. One very significant drawback of Ga ^+^ FIBID is, however, the unavoidable, technique-intrinsic substrate modification that takes place during irradiation. Due to the relatively large mass of Ga ^+^ ions, exposing a sample to a Ga ^+^ FIB results in localized substrate amorphization ^15^, milling ^16^, and ion implantation ^17^. Even though FIBID is reported to have been used to fabricate electrical contacts on robust metals ^18^, it may not be employed for that purpose with fragile materials such as graphene ^19^ or oxides ^20^. Due to the comparatively lighter mass of electrons, and at the cost of a reduced growth rate and (in general) a lower metallic content, FEBID is of great interest to fabricate metallic deposits without significantly affecting the underlying materials.

Some of the most commonly employed precursor materials for the FEBID of metals are trimethyl (methylcyclopentadienyl) platinum, (CH) _3_Pt(CpCH _3_); dicobalt octacarbonyl, Co _2_(CO) _8_; and iron pentacarbonyl and diiron nonacarbonyl, Fe(CO) _5_ and Fe _2_(CO) _9_^10^. Pt-C deposits fabricated by FEBID exhibit room-temperature resistivity values in the order of 10 000 µΩ cm and a metallic content of around 20%, and a relatively high growth rate when compared to other precursor materials; which makes it suitable for the fabrication of protective layers during the preparation of samples for transmission electron microscopy (TEM) experiments ^10^. The poor room-temperature conductivity discourages its usage for the deposition of electrical contacts, although it can be enhanced by subjecting the material to post-treatment methods, such as hydrogen exposure ^21^, and FEB-assisted oxygen purification ^22^. On the other hand, Co-C and Fe-C deposits exhibit much lower room-temperature resistivity (40 µΩ cm and 100 µΩ cm, respectively), but their magnetic behavior might provide unwanted influence on the electric properties of the sample at hand, which also hampers their applicability for their usage as electrical contacts ^23, 24^.

In addition, one other precursor material, widely used for FIBID, is tungsten hexacarbonyl, W(CO) _6_^11^. Its deposition by both Ga ^+^ and He ^+^ FIBID is known to generally yield a material that exhibits metallic behavior at room temperature, and type-II superconductivity below ~5 K ^25, 26^. FEBID of W(CO) _6_ is mostly reported to yield a non-superconducting material with a moderately-metallic electrical response, with reported values of room-temperature resistivity of 3000 µΩ cm ^27^ and 2500 µΩ cm ^28^, and around 4000 µΩ cm at 260 K ^29^. Under specific growth conditions, mostly related to the usage of comparatively high currents, FEBID W-C has also been shown to display superconductivity at low temperatures ^30, 31^. However, using such high FEB currents during irradiation hampers the resolution of the process, yielding deposits with lateral sizes that are typically in the range of several hundreds of nm. In this contribution, we investigate the suitability of the W-C material grown by FEBID of the W(CO) _6_ precursor for its usage in the fabrication of electrical contacts. By using a moderate electron beam current of 1.4 nA and an acceleration voltage of 20 kV, a W-C material with a room-temperature resistivity in the range of 550 µΩ cm can be grown with a remarkable lateral resolution in the order of 45 nm. In the following, the growth conditions employed to obtain a sufficiently-metallic material ( *i.e.*, with a sufficiently high conductivity) are described, and the electrical and compositional characterization studies of the material are presented. Lastly, the applicability of the material is demonstrated in a low-temperature measurement of a superconducting W-C nanowire fabricated by Ga ^+^ FIBID, showing that the contacts are operative from room temperature down to 2 K.

## Methods

The nanofabrication of the W-C electrical contacts was carried out in a commercial Thermo Fisher *Helios 600 Dual Beam* FIB/SEM microscope, fitted with a Ga ^+^ FIB column and a field emission gun electron column, and a gas injection system (GIS) for gaseous precursor delivery. Si/SiO _2_ pieces with titanium pads pre-patterned by OL were used as substrates.

The process chamber of the FIB/SEM microscope had a base pressure in the order of 1 × 10 ^−6^ mbar, which was raised by one order of magnitude during the injection of the precursor material. For each deposition type ( *e.g.*, FEBID and FIBID), the W(CO) _6_ GIS nozzle was positioned 50 µm and 100 µm away from the irradiation point in the in-plane and vertical directions, respectively.

The following parameters were used during deposition of the electrical contacts: electron beam current of 1.4 nA, dwell time of 100 µs, pitch of 7 nm (corresponding to an overlap between consecutive irradiation spots of 60%), and a nominal volume per dose of 8 × 10 ^−6^ nm ^3^ nC ^−1^. The influence of the FEB acceleration voltage was explored at values of 5 kV, 10 kV, 20 kV, and 30 kV. The deposition time of each contact varied depending on its size, but for micron-size contacts, typical deposition times ranged between 2 min and 4 min, decreasing as the acceleration voltage was increased. Under these conditions, the nominal spot size of the FEB was of 11.5 nm.

The electrical characterization of the FEBID W-C material itself also required the fabrication of electrical contacts, for which Pt-C deposited by Ga ^+^ FIBID was chosen as a suitable material. For the FIBID fabrication of the superconducting W-C nanowire, the following parameters were used: ion beam current of 1.5 pA, acceleration voltage of 30 kV, and a nominal volume per dose of 8.3 × 10 ^−2^ nm ^3^ nC ^−1^.

The composition of the contacts was assessed by means of TEM techniques, namely high angle annular dark field (HAADF) imaging and energy dispersive X-Ray spectroscopy (EDS). Both were carried out in a commercial FEI *TITAN Low-Base* instrument. The cross-sectional transversal cuts of the contacts were extracted following conventional lamellae preparation in the FIB/SEM instrument.

The electrical characterization of the contacts was performed both inside and outside the process chamber of the FIB/SEM instrument. The *in-situ* room-temperature electrical measurements were performed using a commercial Kleindiek Nanotechnik microprobe station, a Keithley Instruments 6221 DC current source, and a Keithley Instruments 2182A nanovoltmeter. The low-temperature measurements (down to 2 K) of both the FEBID W-C material itself and of the FIBID W-C test nanowire were performed in a commercial Quantum Design *Physical Property Measurement System 9T* instrument.

## Results

### Deposition

The deposition of the W-C nanowires was performed following conventional FEBID procedures, *i.e.*, with normal FEB incidence and using the set of operating parameters described above. Among them, the value of the dwell time was set to a relatively high value of 100 µs, as previously reported in two of the aforementioned studies ^28, 29^, and taking into account that higher dwell times are expected to favor a more efficient decomposition of the adsorbed W(CO) _6_ molecules, provided there is a sufficient amount of them ^27, 31^. The positioning of the GIS with respect to the irradiation point was found to play a very significant role in the quality of the deposits – if the nozzle is positioned too far away (as it may well be the case if the GIS position is optimized for deposition with the FIB, angled with respect to the FEB in the FIB/SEM microscope), the deposits exhibit a disjointed and fragmented appearance as a consequence of an insufficient amount of precursor being delivered near the irradiation point. It is, therefore, crucial to reposition the GIS nozzle when the irradiation type is changed.

**Figure 1.**
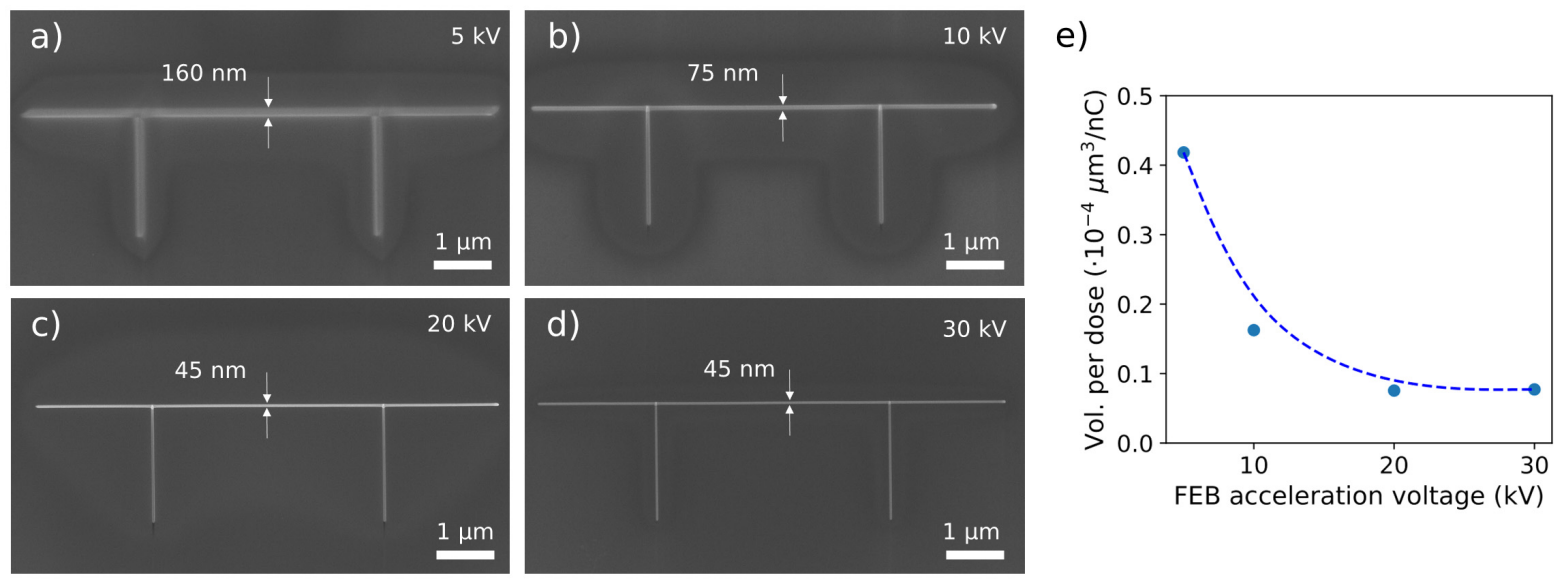
( **a**)–( **d**) Scanning electron microscope images of W-C nanowires grown with a fixed focused electron beam current of 1.4 nA and varying acceleration voltages of 5 kV, 10 kV, 20 kV, and 30 kV. ( **e**) Dependence of the estimated volume per dose with the focused electron beam acceleration voltage. The dashed line is a guide for the eye.

The influence of the FEB acceleration voltage was investigated by growing several 8 µm-long W-C nanowires with distinct values of this operating parameter ( Figures 1a–d) ^32^. With the value of the FEB current fixed at 1.4 nA, increasing the acceleration voltage results in a narrowing in the lateral size of the nanowire, from a width of around 160 nm at 5 kV, down to 45 nm at 20 kV and 30 kV. Lower acceleration voltages require greater irradiation times: in the pictured nanowires ( Figures 1a–d), the deposition times are 5 min 15 s at 5 kV, 3 min 43 s at 10 kV, 2 min 37 s at 20 kV, and 2 min 8 s at 30 kV. However, the volume per dose, defined as the volume of material that can be grown per unit charge, decreases with increasing FEB acceleration voltage ( Figure 1e).

### Electrical characterization

The electrical response of the FEBID W-C contacts was assessed at both room temperature, using the microprobe station mounted in the FIB/SEM instrument, and as a function of temperature down to 2 K.

**Figure 2.**
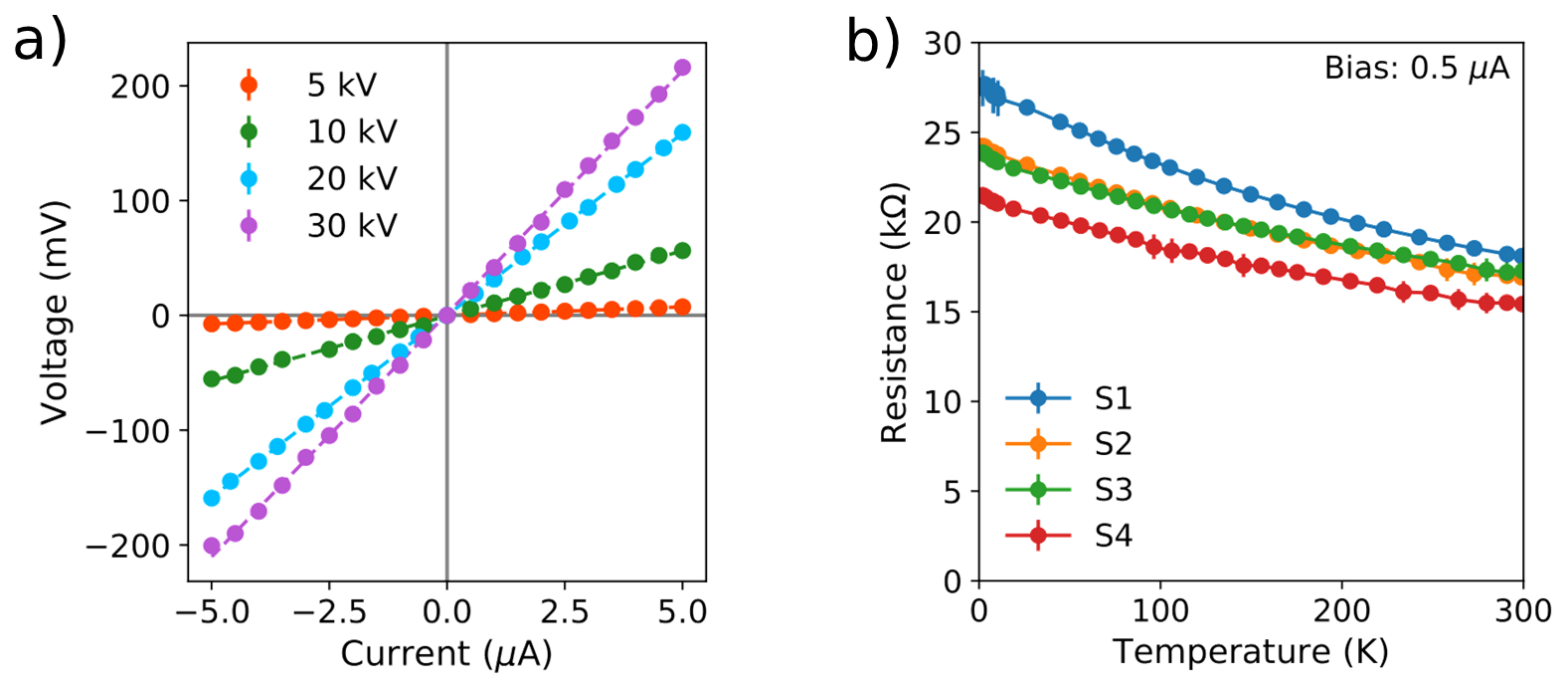
( **a**) Room-temperature *I* − *V* characteristic of W-C nanowires grown by focused electron beam induced deposition using acceleration voltages of 5 kV, 10 kV, 20 kV, and 30 kV. For each acceleration voltage, three nanowires were grown using the same focused electron beam parameters. The represented data in each series correspond to the weighted average of the three measurements. ( **b**) Temperature dependence of the resistance of four equivalent W-C contacts fabricated by focused electron beam induced deposition with an acceleration voltage of 20 kV.

The room-temperature measurements were taken in different sets of nanowires, with each set consisting of three equivalent samples deposited using the same FEB acceleration voltage from among the four previously discussed. All nanowires exhibit a linear, ohmic *I−V* characteristic within the explored current range of ± 5 µA ( Figure 2a). The growth procedure exhibited excellent reproducibility in terms of the obtained electrical resistance, with equivalent nanowires grown using the same deposition parameters showing only reasonably small differences in the measured values of their electrical resistance.

Assessing the resistivity of the material proved challenging due to the discrete nature of the cross-sectioning procedure employed to determine the thickness of the deposits. Despite the apparent reproducibility of the technique observed in the resistance values, different values of thickness are observed in each of the assessed voltages, with values of 70 nm–100 nm for the nanowires grown with an acceleration voltage of 5 kV, 40 nm–60 nm at 10 kV, 30 nm–40 nm at 20 kV, and 25 nm–30 nm at 30 kV. These relatively small differences yield some uncertainty in the calculation of the resistivity. A slight increase of the resistivity with the FEB acceleration voltage is found in the average values: 420±70 µΩ cm at 5 kV, 510 ± 80 µΩ cm at 10 kV, 550 ± 80 µΩ cm at 20 kV, and 700 ± 200 µΩ cm at 30 kV. As anticipated, the FEB acceleration voltage of 20 kV was chosen over the others as a good compromise between acceptable electrical conductivity and good lateral resolution. Thus, the rest of the characterization study was performed on contacts grown with that acceleration voltage only.

The low-temperature study was carried out in four equivalent samples (S1-4), all grown using a FEB acceleration voltage of 20 kV. Within the explored range, 300 K–2 K, the contacts show a negative dependence of the resistance with the temperature ( Figure 2b), and do not exhibit superconducting behavior. The residual resistance ratio, estimated as *R*_300K_/ *R*_2K_, takes values of 0.66, 0.70, 0.72, and 0.72 for the samples S1, S2, S3, and S4, respectively.

### Compositional characterization

**Figure 3.**
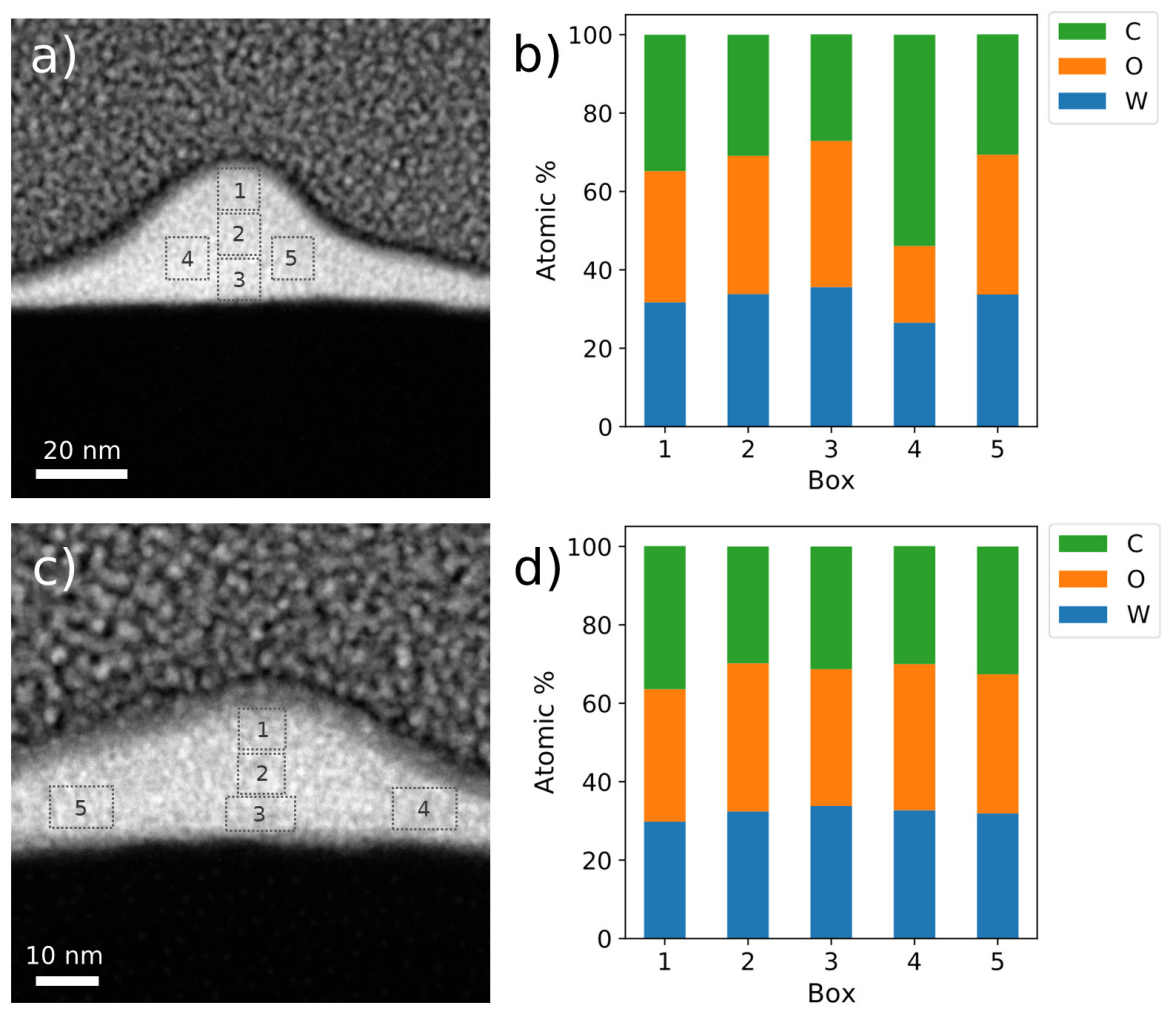
Compositional analysis of two W-C contacts grown by focused electron beam induced deposition. ( **a**) and ( **c**): High angle annular dark field images of samples A and B, respectively, ( **b**) and ( **d**): Energy-dispersive X-ray spectroscopy quantification of the indicated areas for each of these two contacts.

The compositional study was carried out on two equivalent FEBID W-C nanowires, hereafter referred to as A and B. As evidenced by HAADF imaging, the nanowires exhibit a dome-like cross-sectional shape ( Figure 3a and 3c). For sample A, the thickness is 30 nm and the width at half maximum of 40 nm, whereas for sample B, the thickness is 15 nm and the width at half maximum remains at 40 nm. Again, we ascribe the thickness difference to small beam drift and instability effects, mechanical and/or thermal in origin, that take place during growths that take several minutes to complete.

Sample A exhibits a W:C:O ratio of 34:35:31 in terms of atomic percentage, while sample B shows a similar distribution of 32:36:32.

### Contact usage at low temperature

**Figure 4.**
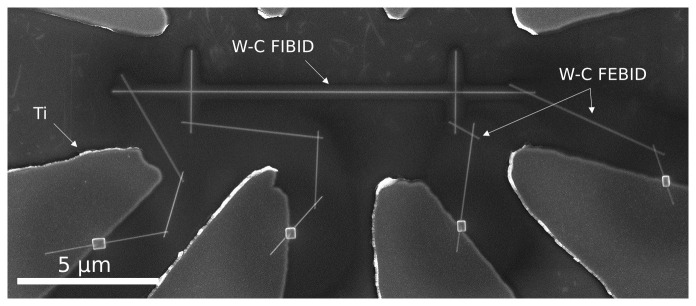
Scanning electron microscopy image of a superconducting W-C nanowire fabricated by Ga ^+^ focused ion beam induced deposition, electrically contacted to Ti pads by W-C contacts grown by focused electron beam induced deposition.

The performance of the FEBID W-C nanowires was put to the test by using them to electrically contact a 10 µm-long, 50 nm-wide W-C nanowire fabricated by Ga ^+^ FIBID to pre-patterned Ti pads on a Si/SiO _2_ substrate ( Figure 4).

**Figure 5.**
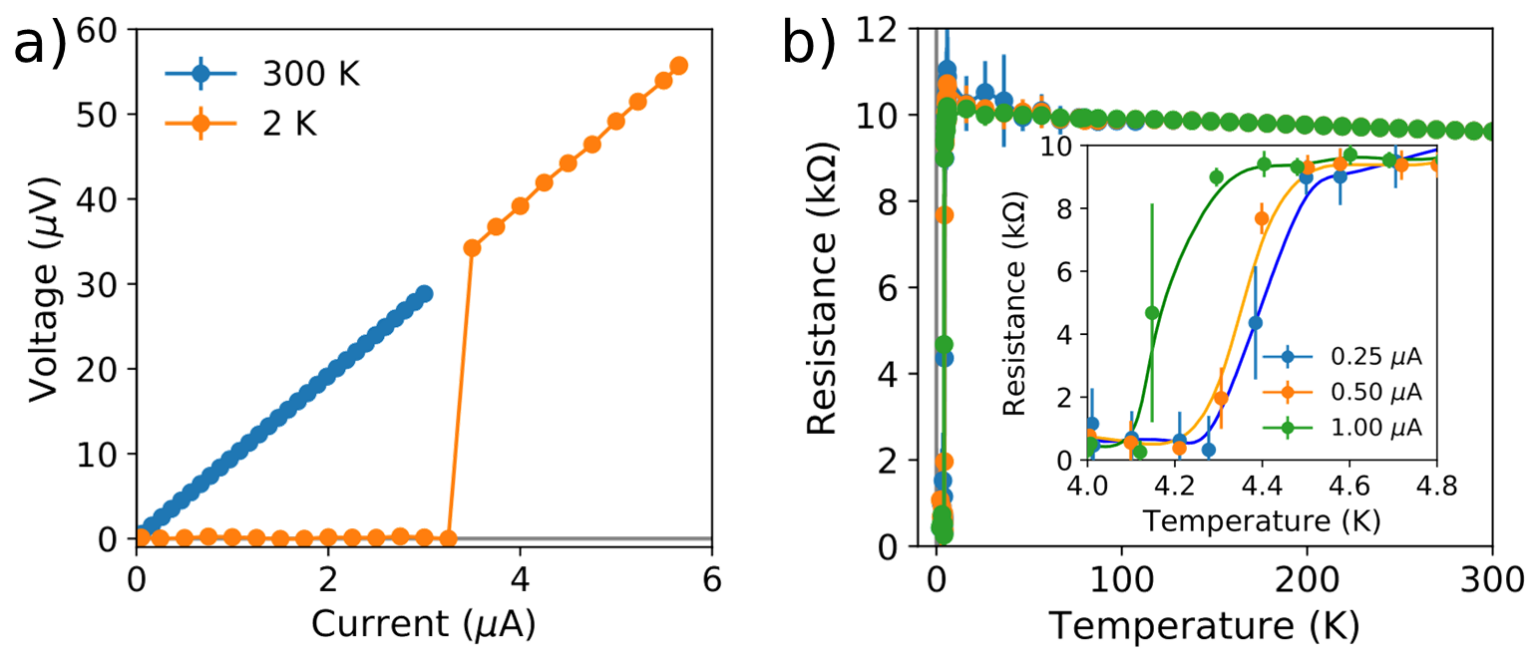
Electrical characterization of a superconducting focused ion beam induced deposition W-C nanowire using W-C contacts fabricated by focused electron beam induced deposition. ( **a**) Voltage dependence with the bias current, at both 300 K and 2 K. The current-induced transition to the normal state can be seen in the latter. ( **b**) Resistance dependence on the temperature, measured using three different bias currents. Inset shows a magnified view of the region where the temperature-induced transition to the superconducting state occurs.

At room temperature, the voltage measured along the nanowire displays a linear dependence with the bias current ( Figure 5a), as expected for the Ga ^+^ FIBID W-C material. With a room-temperature (300 K) resistance of around 10 kΩ, the resistivity of the material can be estimated to take a value around 200 µΩcm, in good agreement with previous reports for this material ^25^. At 2 K, the nanowire is superconducting, and is driven to the normal state when the bias current exceeds a critical value of 3.4 µA.

The temperature-induced transition to the superconducting state is observed at 4.4 K ( Figure 5b).

## Discussion

The dependence of the volume per dose with the FEB acceleration voltage ( Figure 1e) follows a similar trend to that retrieved in the study of FEBID Pt-C ^33^, accounted for by a higher amount of secondary electrons reaching the substrate surface when low voltages are used. After the electrical characterization, the acceleration voltage of 20 kV was deemed as the most appropriate for the purposes of the present study. Since the average thickness of the deposits (retrieved by SEM inspection of cross-sectional cuts) is found to be of 30 nm, the average electron dose required to achieve such a thickness at 20 kV of FEB acceleration voltage equals 4 × 10 ^7^ µC cm ^−2^. For comparison, Blom *et al.* report an electron dose of the order of 10 ^8^ µCcm ^−2^ – 10 ^9^ µCcm ^−2^ for FEBID W-C ^31^, while platinum and cobalt are reportedly grown via FEBID with electron doses in the range of 10 ^5^ µCcm ^−2^ and 10 ^6^ µCcm ^−2^, respectively ^14, 33^.

The 32%–34% of metallic W present in the samples represents a similar value detected in other W-C deposits fabricated by both FEBID and FEBID: W-C fabricated by Ga ^+^ FIBID is reported to show atomic W contents in the 20%–50% range ^25, 28, 34, 35^, and Huth *et al.* report an achieved maximum metallic content of 37% for FEBID W-C ^29^.

The suitability of the contacts is confirmed by the successful characterization of the W-C nanowire fabricated by Ga ^+^ FIBID, where the detected value of critical temperature (4.4 K) is slightly below the commonly reported figure of 4.7 K, but is to be expected for a 50 nm-wide nanowire ^1^.

## Conclusions

We have shown that it is possible to grow high-resolution W-C electrical contacts by FEBID, which represents a relevant alternative to the use of FIBID and allows for avoiding its side effects. Using a FEB acceleration voltage of 20 kV, a FEB current of 1.4 nA, and an electron dose of 0.4 µCµm ^−2^, the deposition procedure yields a W-C material that can be nanopatterned with resolution down to 45 nm, exhibits an average thickness of 30 nm and has a W content of around 30% in terms of atomic composition. With a room-temperature electrical resistivity of 550 µΩcm, the material maintains good conductivity properties down to 2 K, which enables its usage for the electrical characterization of materials across a wide temperature range.

As a proof of concept, we have used these FEBID W-C contacts for the characterization of the superconducting transition in a previously-grown nanowire fabricated by FIBID. These findings open the route for the direct-write growth of high-resolution electrical contacts on ion-sensitive materials such as high-mobility semiconductor nanowires, oxides, 2D materials, and others. In addition, the performed optimization is useful to create direct electrical contacts to nanoSQUIDs, as required in scanning SQUIDs based on direct-write techniques ^36^. It also paves the way towards the fabrication of small-sized Josephson junctions based on superconducting FIBID W-C electrodes connected through these optimized non-superconducting FEBID W-C deposits ^31^.

## Data availability

### Underlying data

Zenodo: Data for manuscript sumitted to Open Research Europe, entitled "Low-resistivity, high-resolution W-C electrical contacts fabricated by direct-write focused electron beam induced deposition". https://doi.org/10.5281/zenodo.6959547 ^32^

This project contains the following underlying data:

- Fig1a.jpg
- Fig1b.jpg
- Fig1c.jpg
- Fig1d.jpg
- Fig1e.txt
- Fig2a_raw.txt (full data set from which the averaged data shown in figure 2a was extracted)
- Fig3a.tif
- Fig3b.txt
- Fig3c.tif
- Fig3d.txt
- Fig4.jpg
- Fig5a.txt
- Fig5b.txt

### Extended data

Zenodo: Data for manuscript sumitted to Open Research Europe, entitled "Low-resistivity, high-resolution W-C electrical contacts fabricated by direct-write focused electron beam induced deposition". https://doi.org/10.5281/zenodo.6959547

This project contains the following extended data:

- Fig2a_averaged.txt

Data are available under the Creative Commons Attribution 4.0 International license (CC-BY 4.0).

## Ethics and consent

Ethical approval and consent were not required.
