# Supplementary material for: Low-resistivity, high-resolution W-C electrical contacts fabricated by direct-write focused electron beam induced deposition
Source: Open Res Eur. 2022 Aug 25;2:102. [Version 1] doi: 10.12688/openreseurope.15000.1 (PMC10446045; doi:10.12688/openreseurope.15000.1)
Supplement: Peer review history

# Peer review: Reviewer response for version 1

**DOI:** 10.21956/openreseurope.16215.r30008

**Published:** 2022-09-27

**Authors:** Ding Zhao

**Affiliations:** 1 Key Laboratory of 3D Micro/Nano Fabrication and Characterization of Zhejiang Province, School of Engineering, Westlake University, Hangzhou, China

The authors investigate the deposition of W-C material with the W(CO) _6 _precursor by FEBID and its usage for electrical contracts. The work is clearly and accurately presented. Sufficient details of methods and analysis have been provided to allow replication by others. The conclusions are adequately supported by the results. So I think the article can be indexed after minor revision.

 I am a little confused about "Lower acceleration voltages require greater irradiation times" in Page 4. As the author mentioned later, more secondary electrons contribute to the electron-matter interaction at lower voltages. So lower voltages should have higher yield and efficiency, i.e. less time, right?

 Did the authors try to pattern dense lines or study the minimal gap between two electrical pads fabricated by their method? This can provide more information for applying their method in the semiconductor field.

 Is it necessary to further purify or change the composition of the W-C deposits to improve its conductivity? Please comment on this. 

Is the study design appropriate and does the work have academic merit?

Yes

Is the work clearly and accurately presented and does it cite the current literature?

Yes

If applicable, is the statistical analysis and its interpretation appropriate?

Yes

Are all the source data underlying the results available to ensure full reproducibility?

Yes

Are the conclusions drawn adequately supported by the results?

Yes

Are sufficient details of methods and analysis provided to allow replication by others?

Yes

Reviewer Expertise:

Electron-beam Lithography; Photonic Devices

I confirm that I have read this submission and believe that I have an appropriate level of expertise to confirm that it is of an acceptable scientific standard.

# Peer review: Reviewer response for version 1

**DOI:** 10.21956/openreseurope.16215.r29980

**Published:** 2022-09-13

**Authors:** Aleksandra Szkudlarek

**Affiliations:** 1 Academic Centre for Materials and Nanotechnology, AGH University of Science and Technology, Kraków, Kraków, Poland

In this work, the authors study the W-C FEBID deposits for fabricating direct maskless electrical contacts at the nanoscale. They present the feasibility of depositing the wires such narrow as 45 nm at 20 kV of beam acceleration voltage, which is the main parameter investigated in this work. To get high metallic content, based on the former studies, the authors have selected high beam current and high dwell time during the deposition process. The elemental and morphological analysis of W-C deposit is shown together with a wide characteristic of its electrical properties.

 The article is a coherent whole and contains the necessary information needed to conduct similar experiments and the set of appropriate references, related to the previous research on this topic. The term resolution usually refers to the ratio of FWHM(deposit)/FWHM(beam). Taking into account the estimated nominal size of the beam, this parameter gets close to 4, which is far from the value of 1, obtained in general in electron-limited regime. To keep the consistency rather the term “ultranarrow” might be more relevant. The technical detail remains what is the definition of the nominal beam size (FWHM, 90% of the total electron flux, etc.) and how it was derived.

 Although the dose can be a reference point for growing the deposit with a certain thickness (while the other parameters are fixed) it cannot be a guideline for volumetric growth. As in FEBIP the other parameters (dwell time, refresh time, beam overlapping, pitch point) will also play a significant role, for example: by keeping exactly the same dose and using relatively short and long times the deposit profile can be very different. At 20 keV can the halo deposit (which is around 2-3 um) play any role? Those aspects were not pointed out in the manuscript.

 Studying the deposit morphology by TEM, the authors have observed for two equivalent lines can have significantly different thicknesses (1:2). This effect was ascribed to the beam instability or drift. This is very unusual behavior unless a major process parameter was changed. Otherwise, it can happen if the lines were deposited sequentially in close vicinity of each other, in the area of depleted precursor.

 The last factor worth considering to include in the conclusions is whether the aging process of those contacts takes a place (due to the oxidation of W), and what are safe current densities limits to work on without observing any W-electromigration or post-annealing by Joule heating.

Is the study design appropriate and does the work have academic merit?

Yes

Is the work clearly and accurately presented and does it cite the current literature?

Yes

If applicable, is the statistical analysis and its interpretation appropriate?

Partly

Are all the source data underlying the results available to ensure full reproducibility?

Yes

Are the conclusions drawn adequately supported by the results?

Partly

Are sufficient details of methods and analysis provided to allow replication by others?

Yes

Reviewer Expertise:

Kinetics of FEBIP

I confirm that I have read this submission and believe that I have an appropriate level of expertise to confirm that it is of an acceptable scientific standard.
